# Factors associated with post‑operative complications in oral carcinoma: Prospective study^☆^^☆☆^

**DOI:** 10.1016/j.bjorl.2025.101610

**Published:** 2025-05-14

**Authors:** Paulo Victor Sola Gimenes, Daniel Abreu Rocha, Marco Aurélio Vamondes Kulcsar, Claudio Roberto Cernea, Leandro Luongo Matos

**Affiliations:** a Universidade de São Paulo (USP), Faculdade de Medicina (FM), Hospital das Clínicas (HC), São Paulo, SP, Brazil; b Universidade de São Paulo (USP), Faculdade de Medicina (FM), Instituto do Câncer do Estado de São Paulo (ICESP), São Paulo, SP, Brazil

**Keywords:** Mouth neoplasms, Postoperative complications, Prognosis

## Abstract

**Objective:**

Patients who require surgery to treat head and neck cancer are at higher risk of perioperative complications and this scenario is not different for patients with malignant tumors of the oral cavity. Thus, the objective of the present study was to identify preoperative prognostic factors related to postoperative complications in the surgical treatment of oral cancer.

**Methods:**

Prospective study with 43 consecutive squamous cell carcinomas of the oral cavity submitted to surgical treatment with curative intent.

**Results:**

Seventeen patients presented postoperative complications until the thirtieth day of follow-up, of which 17.6% required surgical reoperation. The most common complications were suture dehiscence and surgical site infections. Alcohol abuse (p = 0.004), pT4a tumors (p = 0.044), tumors with thickness greater than 10 mm (p = 0.002), patients with mGPS score 1 and 2 (p = 0.027) and flap reconstruction (p < 0.001) were associated with higher rates of postoperative complications. To the multivariate analysis, patients with tumor thickness greater than 10 mm (HR = 11,240; 95% CI 1,052–120,059; p = 0.045 ‒ logistic regression) and reconstructed with myocutaneous flap (HR = 18,415; 95% CI 1,849−183,359; p = 0.013 ‒ logistic regression) had a higher risk of developing postoperative complications.

**Conclusion:**

Tumor thickness greater than 10 mm or use of myocutaneous flaps in the reconstruction were the predictors of risk of postoperative complications in patients with squamous cell carcinoma of the oral cavity.

**Level of evidence:**

Level III.

## Highlights

• The most common complications were suture dehiscence and surgical site infections.
• Tumor thickness >10 mm increased risk of complications.
• Myocutaneous flap linked to higher complication rates.
• High mGPS scores predicted worse postoperative outcomes.

## Introduction

Oral cavity cancer has the resection of the primary tumor associated with selective or therapeutic neck dissection as main treatment modality. The ablations of the primary lesions range from simple partial resections to combined procedures, such as mandibulectomies requiring the need for microsurgical reconstruction with free flaps or pedicled flap rotation.

Patients who require surgery to treat head and neck cancer are at a higher risk of perioperative complications and this scenario is no different for patients with malignant tumors of the oral cavity. Avoiding complications in the postoperative period is a challenge that starts with the identification of patients at higher risk of developing these complications.^1^, ^2^

Many variables are implicated as prognostic factors for complications after surgery, including nutritional status, age, characteristics of the neoplasia and presence of comorbidities. Thus, the objective of this study was to identify preoperative prognostic factors related to postoperative complications in the surgical treatment of oral cancer.^1^, ^2^

## Methods

This is a prospective study, approved by the Research Ethics Committee (protocol 228/14), including 43 consecutive patients with squamous cell carcinoma of the oral cavity submitted to surgical treatment for curative purposes from August 2016 to April 2017. Those patients who underwent salvage surgeries or those with tumors of the lips were excluded.

The data collected were age, gender, primary site, degree of differentiation, alcoholism, smoking, clinical and pathological stage (TNM), tumor thickness, perineural and angiolymphatic invasion, resection margins, extracapsular spread, radiotherapy or adjuvant chemotherapy, Body Mass Index (BMI), patient status by the KPS and ECOG scales, albumin and C-Reactive Protein (CRP) closer to the day of surgery for stratification of the Glasgow Modified Prognostic Score (mGPS), plus the need and type of the flap used in the reconstruction, and the presence of postoperative complications up to the thirtieth day.

The final pathological stage of all patients was reviewed according to the guidelines of the 8th edition of the Cancer Staging Manual of the American Joint Committee on Cancer.^3^

GPS is a prognostic score created in 2008 by the University of Glasgow by McMillan et al.^4^ to evaluate the repercussion of systemic inflammatory status in these patients. It evaluates the patient’s CRP and albumin levels and graduates them according to these values: 0 points ‒ CRP ≤ 10 mg/L and albumin ≥ 35 g/L; 1 point – CRP ≥ 10 mg/L or albumin < 35 g/L; 2 points ‒ CRP > 10 mg/L and albumin <35 g/L. Since the finding of hypoalbuminemia without increase of CRP is rare and that hypoalbuminemia alone does not alter survival, the score was modified and mGPS was created,^5^ which is quite similar to the previous one, and it changes only the graduated group with 1 point: 0 points ‒ CRP ≤ 10 mg/L and albumin ≥35 g/L; 1 point ‒ CRP ≥ 10 mg/L; 2 points ‒ CRP > 10 mg/L and albumin <35 g/L.^4^, ^5^

Karnofsky performance scale is a classification developed by David A. Karnofsky in his 1948 article on the use of nitrogen mustard in the palliative treatment of carcinomas. This graduated scale of 0–100 points evaluates the patient’s ability in developing activities through 11 different measures: 0 points (death); 10 points (dying, death process in rapid progression); 20 points (very weak, hospitalization required); 30 points (very incapable; indicated hospitalization, although death is not imminent); 40 points (unable; need of special care and assistance); 50 points (need of substantial assistance and frequent medical care); 60 points (need of occasional assistance but still able to provide most of your activities in need of active support treatment); 70 points (able to take care of oneself; unable to carry out normal activities or perform active work); 80 points (some signs or symptoms of disease with effort); 90 points (capable of normal activities, small signs and symptoms); 100 points (normal; no complaint or evidence of illness).^6^

The ECOG scale was developed by the Eastern Cooperative Oncology Group (ECOG), now part of the ECOG-ACRIN Cancer Research Group, and published in 1982.^7^ It describes the level of functionality of a patient in terms of ability to care of oneself and physical ability to perform daily activities (walking, working, etc.) and measured in 5 categories: 0 (fully active, capable of performing all of the activities without restriction); 1 (restriction to strict physical activities, capable of light work and sedentary nature, restricted to the bed only in the nocturnal periods of sleep); 2 (able to perform all self-care but unable to perform any work activity; confined to the bed or chair less than 50% of the hours the patient is awake); 3 (able to perform only limited self-care, confined to the bed or chair more than 50% of the hours the patient is awake); 4 (completely unable to perform basic self-care, completely confined to the bed or chair).

The nutritional deficit is commonly evaluated by the Body Mass Index (BMI). This index is obtained by the product of weight division by squared height of a person and it is used by the World Health Organization to classify individuals in BMI < 18.5 kg/m^2^ ‒ underweight; BMI ≥ 18.5 up to 24.9 kg/m^2^ ‒ normal weight; BMI ≥ 25.0–29.9 kg/m^2^ ‒ overweight; BMI ≥ 30 up to 34.9 kg/m^2^ ‒ class I obesity; BMI ≥ 35.0 up to 39.9 kg/m^2^ ‒ class II obesity; BMI ≥ 40 kg/m^2^ ‒ class III obesity.

The primary outcome of the study was to identify risk factors for postoperative complications by the thirtieth day after the procedure. For this, the complications were first classified in a binary way, that is, complications of any nature, clinical or surgical. Complications were categorized into major and minor complications. Larger complications were those requiring reoperation in a surgical center and under anesthesia. Minor complications were those treated conservatively, that is, without the need for further repair surgery. Conservative treatment comprised dressings, small drains or debridement, and medication use.^8^ When one complication led to another, only the latter was considered in an attempt to represent the actual outcome in an individual patient. For example, if a dehiscence resulted in an orocutaneous fistula, then the fistula was ultimately considered as the complication. Ischemic complications, such as partial or total loss of the flap were analyzed separately, independently of the other complications associated with the ischemic event. For example, if a partial loss led to dehiscence and fistula, then they considered “partial loss” and “fistula”.^8^

### Statistical analysis

The values obtained by the study of each quantitative variable of parametric distribution were organized and described through the mean, standard deviation, minimum and maximum. For the qualitative variable, absolute and relative frequencies were used. Frequency comparisons were performed using Fisher’s exact test. Variables with p < 0.10 were selected for multivariate analysis using the logistic regression model with Hazard Ratio (HR) and 95% Confidence Intervals (95% CI). In all analyses, the statistical program SPSS version 17.0 (SPSS Inc; Ilinois, USA) was used and in all comparisons a level of statistical significance was adopted below 5% (p ≤ 0.05).

## Results

The sample consisted of 43 patients, between the sixth and seventh decades of life, predominantly male. The most frequent subsets of the primary lesions were oral tongue and floor of the mouth (69.7%), with advanced stages being the most incident (pT3 or pT4a with lymph node metastasis). The complete descriptive data are described in Table 1.

**Table 1:** Descriptive data of patients included in the study.

Variables	Result
**Demographic data**	
Male	33 (76.7%)
Age (years)	60.4 ± 11.5 (31–87)
Primary site	
Oral tongue	17 (39.5%)
Floor of the mouth	13 (30.2%)
Buccal mucosa	6 (14%)
Lower alveolar ridge	4 (9.3%)
Retromolar area	2 (4.7%)
Hard palate	1 (2.3%)
Smoking	32 (74.4%)
Etilism	23 (53.5%)
**Anatomy-pathological data**	
Free resection margins	34 (79.1%)
Degree of differentiation	
Well differentiated	13 (30.2%)
Moderately differentiated	20 (46.5%)
Poorly differentiated	10 (23.3%)
Perineural invasion	21 (48.8%)
Angiolymphatic invasion	11 (25.6%)
pT	
pT1	7 (16.3%)
pT2	7 (16.3%)
pT3	15 (34.9%)
pT4a	14 (32.6%)
pN	
pN0	18 (41.9%)
pN1	7 (16.3%)
pN2a	1 (2.3%)
pN2b	4 (9.3%)
pN2c	3 (7.0%)
pN3b	10 (23.3%)
Tumor size	38.1 ± 22.6 (4–90) mm
Tumor thickness^a^	20.1 ± 14.6 (1–60) mm
Tumor thickness >10 mm (n = 39)	26 (60.5%)
Extracapsular spread (n = 25)	12 (48.0%)

a Mean ± Standard Deviation (minimum ‒ maximum).

The modified Glasgow Prognostic Score (mGPS) was the predominance of individuals with scores 1 (CRP > 10 mg/dL) or 2 (CRP > 10 mg/dL and albumin <3.5 mg/dL) in 53.5% of cases. There was also a predominance of patients with good performance status and eutrophic. Moreover, most of the patients required myocutaneous flap for reconstruction of the surgical defect. Seventeen patients presented postoperative complications up to the thirtieth day of follow-up, and 17.6% of them required surgical reoperation. The most common complications were suture dehiscence and surgical site infections. The full description of the prognostic factors and postoperative complications are detailed in Table 2.

**Table 2:** Prognostic factors and postoperative complications.

Variables	Result
**Prognostic factors**	
Glasgow Prognostic Score Modified (mGPS)	
Score 0 (CRP ≤ 10 mg/dL and albumin ≥ 3.5 mg/dL)	20 (46.5%)
Score 1 (CRP > 10 mg/dL)	19 (44.2%)
Score 2 (CRP > 10 mg/dL and albumin < 3.5 mg/dL)	4 (9.3%)
ECOG	
0	7 (16.3%)
1	32 (74.4%)
2	3 (7.0%)
3	1 (2.3%)
KPS	
50	1 (2.3%)
70	2 (4.7%)
80	18 (41.9%)
90	19 (44.2%)
100	3 (7.0%)
Body Mass Index (BMI)^a^	26.0 ± 7.5 (16.8–53.1) kg/m^2^
Body Mass Index < 19.5 kg/m^2^	4 (9.3%)
Reconstruction with flap	24 (55.8%)
Free flap	13 (30.2%)
Pediculated flap	11 (25.6%)
**Postoperative complications**	
General complication	17 (39.5%)
Major complications	3 (7.0%)
Minor complications	9 (20.9%)
Description of complications:	
Suture dehiscence	6 (35.3%)
Surgical site infection	4 (23.5%)
Orocutaneous fistula	3 (17.6%)
Cervical hematoma	3 (17.6%)
Partial flap loss	2 (11.8%)
Pneumonia	1 (5.9%)
Venous thrombosis	1 (5.9%)

a Average ± Standard Deviation (minimum ‒ maximum).

Alcohol abuse (p = 0.044), pT4a tumors (p = 0.044), tumors with thickness greater than 10 mm (p = 0.002), patients with mGPS scores 1 and 2 (p = 0.027) and flap reconstruction (p < 0.05) 0.001) were associated with higher rates of postoperative complications in patients submitted to surgical treatment for squamous cell carcinoma of the oral cavity (Fisher’s exact test) ‒ Table 3.

**Table 3:** Analysis of factors related to the development of complications.

Variable		Complication rate	p-value (Fisher exact)
Smoking	No	2/11 (18.2%)	0.154
	Yes	15/32 (46.9%)	
Alcohol abuse	No	3/20 (15.0%)	**0.004**
	Yes	14/23 (60.9%)	
Margins	Free	13/34 (38.2%)	1.000
	Positive	4/9 (44.4%)	
pT	pT1, pT2, pT3	8/29 (27.6%)	**0.044**
	pT4a	9/14 (64.3%)	
Tumor thickness	≤10 mm	1/13 (7.7%)	**0.002**
	>10 mm	16/26 (61.5%)	
pN	pN0 pN+	5/18 (27.8%)	0.219
		12/25 (28.0%)	
mGPS	Score 0	4/20 (20.0%)	0.027
	Score 1	12/19 (63.2%)	
	Score 2	1/4 (25.0%)	
ECOG	0/1	17/39 (43.6%)	0.140
	2/3	0/4 (0.0%)	
KPS	>70	17/40 (42.5%)	0.266
	≤70	0/3 (0.0%)	
BMI	≥18.5 kg/m^2^	14/39 (35.9%)	0.284
	<18.5 kg/m^2^	3/4 (75.0%)	
Reconstruction with flap	No	1/19 (5.3%)	**<0.001**
	Yes	16/24 (66.7%)	

To the multivariate analysis, patients with tumor thickness greater than 10 mm (HR = 11,240; 95% CI 1,052–120,059; p = 0.045 ‒ logistic regression) and reconstructed with myocutaneous flap (HR = 18,415; 95% CI 1,849‒183,359; p = 0.013 ‒ logistic regression) had a higher risk of developing postoperative complications ‒ Table 4.

**Table 4:** Multivariate analysis of factors determining postoperative complications.

Variable	HR	95% CI	p^a^
Alcohol abuse	6.448	0.941‒44.200	0.058
pT4a	0.309	0.029‒3.333	0.333
Thickness >10 mm	**11.240**	**1.052‒120.059**	**0.045**
mGPS scores 1 and 2	1.603	0.206‒12.476	0.652
Reconstruction with flap	**18.415**	**1.849‒183.359**	**0.013**

a Logistic regression.

## Discussion

This study evaluated the influence of clinical, pathological and prognostic factors on complications up to the 30th postoperative day in patients with squamous cell carcinoma of the oral cavity. It was identified that tumor thickness greater than 10 mm and the use of myocutaneous flap in the reconstruction were independent risk factors for postoperative complications.

For decades, the tumor thickness was associated with a worse prognosis, with lower survival and higher rates of treatment failure. When this value is equal or greater than 5 mm, the risk for cervical metastasis is higher and it is considered a “discerning point”. Invasive depth greater than 10 mm is considered as deeply invasive cancer, which led to the American Joint Committee on Cancer (AJCC) incorporating depth of invasion as one of the criteria to be evaluated in the T classification of the new TNM.^9^, ^10^, ^11^ In this study, for the first time in the literature, a positive relationship between thickness greater than 10 mm and a greater chance of postoperative complications was identified. Possibly, thicker tumors require larger resections and, therefore, they are associated with higher rates of complications.

It was also identified that 55.8% of the patients requiring myocutaneous flap (pedicled or microsurgical) presented postoperative complications, as corroborated by the literature. In these patients, complication rates range from 13% to 65%.^12^ Furthermore, the literature is not consensual about the definition of complications, and there is a wide variety of scenarios, which contributes to such a high dispersion.

Operative wound dehiscence is a common complication in head and neck surgeries, especially in patients who underwent radiation and reconstruction with flap.^13^ In our analysis, 17.6% of the patient’s required reoperation and six of these patients (35.3%) were due to suture dehiscence. When the reconstruction of the oral cavity with myocutaneous flap is performed, the most feared complication is the orocutaneous or pharyngocutaneous fistula, present in about 10.7%–40% of the cases.^12^ In this study, there was a fistula rate of 17.6%, regardless of the type of flap used, and only one case required reoperation.

In the case of microsurgical reconstruction, the main complications that required revision of the vascular anastomosis occurred in about 16.2%.^14^ against 5.9% in our series. According to this author, total dehiscence of the flap occurred in 4.9% of the cases, with 95.1% of the flap survival after surgical revision. Likewise, patients with these complications appear to have reduced survival (specific survival of approximately 60% at 5-years for stages III and IV).^14^

Patient-related factors such as nutritional status, performance status, and level of systemic inflammatory response are associated with the prognosis of patients with head and neck cancer.^6^, ^7^ Functional status assessments are often employed to complement medical information and characterize the impact of the disease on the patient. Loss of function is usually related to the cumulative physical, physiological, and psychological effects of the disease process.^6^, ^7^ In cancer therapy clinical trials, performance status has been shown to be an important predictor of response to therapy and surviva.^14^

In this study, alcohol abuse and elevated mGPS scores were associated with postoperative complications.

Patients who do not interrupt the use of alcoholic behavior tend to have more complications associated with the therapy and an increase in the risk of multiple primary tumors.^2^ In this study, also corroborating what is found in the literature, we find that alcoholism as an associated factor to postoperative complications.

In the mGPS analysis, individuals with scores 1 (CRP > 10 mg/dL) and 2 (CRP > 10 mg/dL and albumin <3.5 mg/dL) were predominant. mGPS is considered an independent prognostic factor for cancer patients and its use has already been reported for patients with operable disease, patients undergoing chemotherapy/radiotherapy, and for inoperable cases. An association of 15 studies with more than 2,000 patients demonstrated that high mGPS scores are associated with greater weight loss, poor performance status, increased comorbidities, and treatment complications.^5^ Our study corroborates data found in the literature, with high rates of postoperative complications in these patients.

The main virtue of this study was the prospective model, but the limitation was the reduced sample. Thus, the study continues with the inclusion of new patients.

## Conclusion

Tumor thickness greater than 10 mm or use of myocutaneous flaps in the reconstruction were the predictive factors of risk of postoperative complications in patients with squamous cell carcinoma of the oral cavity.

## Declaration of competing interest

The authors declare no conflicts of interest.
